# Chronic circadian misalignment in military personnel: a potential contributor to neuroinflammation risk

**DOI:** 10.3389/fnmol.2026.1960208

**Published:** 2026-10-16

**Authors:** Attila Novak, Timea Teglas

**Affiliations:** 1 Scientific Research Centre of the Hungarian Defence Forces Transformation Command, Budapest, Hungary; 2 Doctoral College Health Sciences Division, Semmelweis University, Budapest, Hungary; 3 Department of Sports Medicine and Digital Health, Faculty of Health and Sports Sciences, Széchenyi István University, Győr, Hungary

**Keywords:** circadian rhythm, cognitive dysfunction, military, mood disturbances, neuroinflammation

## Abstract

Circadian rhythm disruption is increasingly recognized as a key contributor to neurological disorders; however, its role is often viewed as a secondary consequence rather than a primary driver of brain dysfunction. We argue that chronic circadian misalignment should be reconceptualized as a potential, modifiable contributor to neuroinflammatory risk, rather than viewed solely as a secondary consequence. Military personnel represent a unique and underutilized human model in this context, as they are chronically exposed to predictable circadian stressors, including irregular training schedules, overseas travel, sleep restriction, and pre-operational stress. At the molecular level, circadian misalignment disrupts core clock gene regulation (e.g., CLOCK, BMAL1), leading to downstream alterations in mitochondrial function and redox homeostasis. These changes promote a sustained pro-inflammatory milieu, impair neuroimmune balance, and compromise synaptic plasticity and blood–brain barrier integrity. We propose that these mechanisms collectively increase vulnerability to cognitive dysfunction and mood disturbances, even in otherwise healthy and physically fit populations. Thus, circadian misalignment in military personnel is not merely a byproduct of performance demands, but a biologically plausible contributor to brain dysfunction that warrants targeted intervention and further mechanistic study. Reframing circadian disruption as a potential, modifiable neurobiological risk factor has significant implications for both research and practice.

## Graphical abstract

**Figure:**
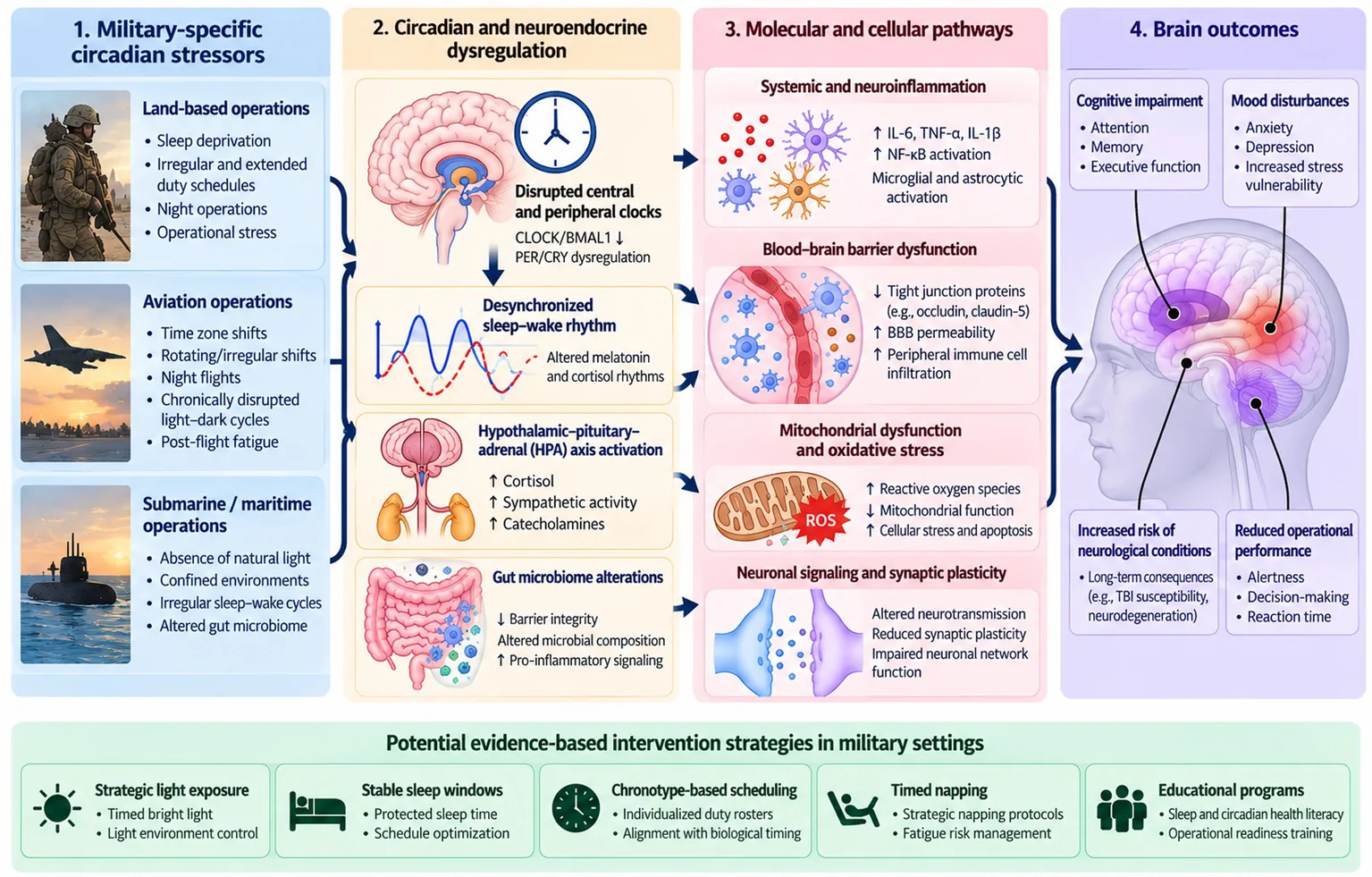

## Introduction

The circadian rhythm is an endogenous biological timing system that regulates a wide range of physiological and behavioral processes over an approximately 24-h cycle (Fishbein et al., 2021). This internal timing mechanism enables organisms to anticipate predictable environmental changes associated with the Earth’s light–dark cycle, thereby optimizing physiological functions such as the sleep–wake cycle, hormone secretion, body temperature, metabolism, immune responses, and cognitive performance. In mammals, the central circadian pacemaker is located in the suprachiasmatic nucleus (SCN) of the anterior hypothalamus, which receives direct photic input from intrinsically photosensitive retinal ganglion cells through the retinohypothalamic tract. Light serves as the primary environmental cue (zeitgeber time, ZT), synchronizing the endogenous clock with the external day-night cycle (von Gall, 2022). At the molecular level, circadian rhythms are generated by a complex transcriptional-translational feedback loop involving core clock genes, including CLOCK, BMAL1, PER1-3, and CRY1-2. These genes regulate rhythmic expression of thousands of downstream genes across multiple tissues, allowing biological processes to occur at the most appropriate time of day. In addition to the central pacemaker, peripheral clocks are present in nearly every organ, including the liver, heart, skeletal muscle, adipose tissue, and brain, coordinating local physiological functions while remaining synchronized with the SCN (Bautista et al., 2025). Circadian rhythms regulate nearly every aspect of mammalian physiology, coordinating sleep–wake cycles, endocrine function, metabolism, immune responses, and neuronal activity through synchronized molecular clocks. Circadian misalignment refers specifically to a mismatch between the endogenous circadian phase (e.g., core body temperature, melatonin, or cortisol rhythms) and the imposed sleep–wake or work schedule, and is properly demonstrated only when circadian phase markers are directly assessed (Wescott et al., 2025). Sleep restriction or deprivation refers to a reduction in total sleep time or complete sleep loss, which can occur with or without a shift in circadian phase and can independently produce inflammatory and cognitive effects (Wüst et al., 2024; Crowley et al., 2024). Night work, shift work, and irregular scheduling describe occupational exposures that frequently, but do not necessarily, produce circadian misalignment. Operational stress refers to psychological or physiological strain associated with military duties, which may co-occur with but is mechanistically distinct from circadian disruption. Altered light exposure (e.g., nocturnal light, absence of natural light) is a zeitgeber-level exposure that can shift circadian phase but is not equivalent to misalignment itself (Ashton et al., 2022).

While transient circadian disruption is a common feature of modern lifestyles, growing evidence suggests that chronic circadian misalignment produces persistent biological alterations that extend well beyond sleep disturbances (Fishbein et al., 2021; Schrader et al., 2024). Recent studies indicate that prolonged disruption of circadian homeostasis contributes to oxidative stress, mitochondrial dysfunction, immune dysregulation, and impaired synaptic plasticity, all of which are recognized contributors to neurological disease (Lodovichi and Ratto, 2023; Xu et al., 2023). Traditionally, circadian disruption has largely been regarded as a secondary consequence of neurological disorders or irregular work schedules. However, accumulating evidence suggests that chronic circadian misalignment may be associated with pathological processes involving altered molecular clock signaling, impaired neuroimmune homeostasis, and low-grade inflammation (Ding et al., 2024; Logan and McClung, 2019; Xu et al., 2023). Experimental studies demonstrate that dysregulation of core clock genes, including CLOCK and BMAL1, affects mitochondrial metabolism, reactive oxygen species (ROS) production, inflammatory signaling, and blood–brain barrier integrity, suggesting that circadian dysfunction may represent an upstream regulator of neurobiological resilience rather than merely a downstream manifestation of disease *in vivo* (Eum et al., 2023; Musiek and Holtzman, 2016; Schurhoff and Toborek, 2023; Teglas et al., 2023, 2024).

The blood–brain barrier (BBB) is a highly specialized neurovascular interface that maintains central nervous system homeostasis by regulating the transport of molecules between the circulation and brain tissue. Disruption of BBB integrity increases vascular permeability and facilitates infiltration of peripheral immune mediators, including pro-inflammatory cytokines, thereby promoting microglial activation and neuroinflammatory responses (Profaci et al., 2020; Varatharaj and Galea, 2017). Consequently, BBB dysfunction is considered a key mechanism linking systemic inflammation, environmental stressors, and neurological alterations affecting cognition and mood. Importantly, preliminary evidence from military populations provides some support for an association between severe operational stress and altered BBB permeability. In soldiers undergoing intensive combat training, circulating levels of S100B, a marker commonly used to indicate altered BBB permeability, were increased (Li et al., 2014). Similarly, a study of U.S. Marines undergoing Survival, Evasion, Resistance, and Escape (SERE) training reported changes in S100B alongside alterations in inflammatory and stress-related biomarkers, suggesting that severe physical and psychological stress may affect BBB-related processes in military personnel (Varanoske et al., 2022). However, these studies did not specifically assess circadian misalignment, and therefore do not establish a direct causal relationship between circadian disruption and BBB dysfunction. Rather, they provide military-specific human evidence supporting BBB alterations as a potential component of the broader physiological response to severe operational stress.

Mitochondria play a central role in maintaining neuronal homeostasis by regulating adenosine triphosphate (ATP) production, calcium signaling, redox homeostasis, and innate immune responses (Missiroli et al., 2020). Mitochondrial dysfunction leads to excessive production of mitochondrial reactive oxygen species (mtROS), impaired oxidative phosphorylation, and the release of mitochondrial damage-associated molecular patterns (mtDAMPs), which activate microglia and promote neuroinflammatory signaling (Marchi et al., 2023). Furthermore, mtROS can stimulate NLRP3 inflammasome activation and NF-κB signaling, thereby increasing the production of pro-inflammatory cytokines, including interleukins (IL-1β, IL-6) and tumor necrosis factor alpha (TNF-α) (Qin et al., 2024). Consequently, mitochondrial dysfunction and oxidative stress are increasingly recognized as key drivers of chronic neuroinflammation and the associated cognitive and affective impairments (Hamzeh et al., 2023).

Growing evidence suggests that disruption of the circadian rhythm can adversely affect both mood and cognitive function (Cajochen and Schmidt, 2025; Dollish et al., 2024). Circadian misalignment has been associated with an increased risk of depression and bipolar disorder, as well as impairments in attention, learning, memory, and executive functioning. Recent reviews indicate that maintaining circadian synchronization may be important for preserving mental health and optimal cognitive performance (McCarthy et al., 2022).

## Circadian disruption as an emerging neurobiological risk factor

Military personnel represent a particularly valuable yet underutilized human model for investigating these mechanisms. Unlike many civilian populations, military service routinely exposes individuals to multiple, predictable sources of circadian disruption, including shift work, prolonged wakefulness, night operations, transmeridian travel, operational stress, and irregular sleep schedules (Good et al., 2020; Mysliwiec et al., 2016). Importantly, these stressors often occur simultaneously and repeatedly throughout military careers, creating sustained circadian misalignment despite otherwise high levels of physical fitness and medical screening (Good et al., 2020; Mysliwiec et al., 2016). Furthermore, emerging evidence suggests that chronic circadian disruption may compromise operational performance by affecting cognitive flexibility, executive function, decision-making, emotional regulation, and stress resilience (Good et al., 2020; Mysliwiec et al., 2016; Walker et al., 2020). These functional impairments are increasingly hypothesized to reflect molecular mechanisms involving neuroinflammation rather than sleep loss alone; this link is a translational inference drawn mainly from preclinical and experimental evidence, as direct evidence of neuroinflammation in operationally impaired military personnel is lacking (Lowe et al., 2017; Yuan and Wang, 2025). Consequently, circadian misalignment should not simply be considered an occupational inconvenience but rather a biologically meaningful factor that may influence both short-term operational readiness and long-term neurological health. Importantly, these military-specific circadian stressors may engage partially distinct molecular pathways that interact and potentially converge on shared downstream processes, including inflammatory signaling and neuroinflammation.

In this mini-review, we synthesize current evidence supporting the concept that chronic circadian misalignment is a potential, modifiable contributor to neuroinflammatory risk in military personnel. We discuss how military-specific circadian stressors disrupt molecular clock regulation and contribute to neuroinflammatory processes across land-based, aviation, and submarine military personnel. Finally, we highlight the implications of reframing circadian disruption as a potential neurobiological risk factor and discuss potential intervention strategies to preserve brain health and optimize operational performance in military populations.

## Circadian disruption in military personnel and linking neuroinflammatory mechanisms

### Circadian challenges in land-based military personnel

Land-based military operations expose personnel to multiple environmental and operational stressors that disrupt circadian rhythms and adversely affect overall operational readiness. A recent study in military cadets demonstrated that total sleep deprivation disrupted circadian rhythmicity, reduced cognitive performance, and altered mood. Importantly, nocturnal light exposure transiently improved alertness but simultaneously perturbed circadian rhythmicity, suggesting that nocturnal light exposure may have divergent effects on short-term alertness and circadian regulation (Skálová et al., 2026). In a study of U.S. Air Force T-6A Texan II instructor pilots, increased serum levels of proinflammatory cytokines, including IL-6 and TNF-α, were associated with elevated fatigue during operational flight duties (Damato et al., 2022). These findings demonstrate an association between elevated circulating pro-inflammatory cytokines and fatigue during operational flight duties (Damato et al., 2022). Importantly, experimental sleep deprivation activates microglia and astrocytes, increases the production of pro-inflammatory cytokines (particularly IL-6, TNF-α, and IL-1β), promotes oxidative stress, and impairs synaptic plasticity. Extending these neuroinflammatory processes to explain the attention, memory, executive function, and emotional regulation deficits observed in sleep-deprived humans is a translational inference rather than a directly demonstrated mechanism, since neuroinflammation is not typically assessed in the same human cohorts in which cognitive and mood outcomes are measured (Yuan and Wang, 2025). A systematic review of randomized controlled trials in healthy young adults demonstrated that both total and partial sleep deprivation consistently impair psychomotor vigilance, attention, executive functioning, and affective state. These findings indicate that even short-term sleep loss produces measurable reductions in cognitive performance and mood in otherwise healthy individuals (Griggs et al., 2022).

Interestingly, a feasibility study in 246 active-duty U.S. Army personnel demonstrated that a mobile health platform (AIRE) could successfully monitor sleep, fatigue, behavioral health, and circadian challenges during field exercises, highlighting the potential of digital technologies to support circadian health and operational readiness in military settings (Germain et al., 2023). In a controlled study involving active-duty military personnel, perception–action coupling performance was significantly influenced by both time of day and simulated military operational stress. Performance deteriorated during circadian low points and under operational stress, with the combined effects exceeding those of either factor alone, indicating that circadian timing substantially modulates cognitive-motor function during military operations (LaGoy et al., 2022). Similar findings have been reported in controlled civilian studies, demonstrating that circadian phase significantly modulates cognitive and psychomotor performance (Lee et al., 2009). Acute psychological stress activates the hypothalamic–pituitary–adrenal (HPA) axis and induces an inflammatory response characterized by increases in circulating inflammatory mediators, including IL-6 and TNF-α (Marsland et al., 2017). A recent meta-analysis of human experimental studies found that several consecutive nights of partial sleep deprivation—rather than circadian phase misalignment, which was not assessed—significantly increased circulating IL-6 and C-reactive protein concentrations, suggesting that sustained sleep loss alone can induce a systemic inflammatory response that may contribute to neuroinflammation and subsequent cognitive and emotional dysfunction (Ballesio et al., 2025). Because these are systemic, peripheral inflammatory markers, this finding demonstrates biological plausibility rather than direct evidence of central neuroinflammation; sustained peripheral inflammation may nonetheless contribute to neuroinflammatory processes and subsequent cognitive and emotional dysfunction via humoral and neural immune-to-brain signaling pathways. When circadian phase is additionally misaligned with the sleep–wake schedule, cortisol rhythmicity and immune regulation may be further altered, potentially exacerbating these stress-related cognitive deficits (Dantzer, 2017).

### Circadian stress and operational demands in military aviation personnel

Military fighter pilots are routinely exposed to a combination of distinct circadian-relevant stressors, including circadian phase misalignment, irregular work schedules, sleep restriction, and high operational demands, although these exposures are rarely disentangled in the available literature. In a recent metabolomic study of Brazilian A-29 fighter pilots, acute flight missions—an operational stress exposure in which circadian phase was not directly assessed—induced measurable alterations in immune cell counts and energy metabolism, with experienced pilots exhibiting greater metabolic adaptation than trainees, suggesting that repeated operational exposure elicits physiological adaptations to chronic occupational stress (Verissimo França de Oliveira et al., 2025). Varma et al. (2025) previously described that Air Traffic Control personnel from the Australian Defence Force reported substantial impacts on sleep, family interactions, and social activities resulting from operational requirements, including irregular duty schedules, shift work, and sustained mental demands (Varma et al., 2025). A recent nested case–control study in Danish military personnel reported that pilots had a higher incidence of prostate cancer than non-flying military controls. The authors proposed that repeated circadian disruption associated with transmeridian flights, together with occupational exposures such as ionizing radiation and prolonged sedentary work, may contribute to this increased risk (Pedersen and Hansen, 2026). Although circadian disruption was not directly measured in the Danish military pilot study, repeated transmeridian flights are recognized as a source of chronic circadian misalignment. Experimental and clinical studies indicate that circadian disruption promotes persistent activation of inflammatory pathways, including elevated production of IL-6 and TNF-α and activation of the nuclear factor kappa-light-chain-enhancer of activated B cells (NF-κB) signaling; these findings directly demonstrate systemic inflammation, while central neuroinflammation is inferred from mechanistic pathways, such as cytokine signaling across the blood–brain barrier, rather than measured directly in most of these studies. These inflammatory mechanisms have been implicated not only in cognitive and mood disturbances but also in cancer development through immune dysregulation, oxidative stress, and impaired DNA repair (Bailey et al., 2014; Froy and Weintraub, 2025; Meyer et al., 2024). Research involving female U. S. military aviation officers identifies night operations, irregular duty schedules, and transmeridian flights as common occupational exposures. However, circadian phase was not directly measured in this study; these exposures are recognized correlates of circadian misalignment in military aviation (Stark et al., 2024). Although the primary focus was reproductive health, the findings reinforce the need to consider circadian-relevant occupational exposures as a systemic health risk in military personnel.

### Circadian disruption in maritime or submarine military operations

Submarine deployment represents a unique military environment characterized by several co-occurring but mechanistically distinct exposures: circadian phase misalignment, sleep loss, and the absence of natural light cues. In submariners, these combined stressors were associated with alterations in gut microbiota composition and mood, with microbiome changes correlating with fatigue; because these exposures were not disentangled in this study, the observed effects cannot be attributed specifically to circadian misalignment rather than to sleep loss or light deprivation alone (Liechty et al., 2026). Similar findings have been reported in civilian studies demonstrating that circadian misalignment disrupts the composition and rhythmicity of the gut microbiota, thereby altering microbial metabolite production and promoting systemic inflammation (Mashaqi and Gozal, 2020; Withrow et al., 2021). These immune changes, characterized by increased pro-inflammatory cytokine signaling and impaired gut barrier integrity, are hypothesized to facilitate gut-brain communication and contribute to neuroinflammatory processes implicated in fatigue, mood disturbances, and cognitive dysfunction; extending these civilian mechanistic findings to military gut-brain-neuroinflammation outcomes is a translational inference, as it has not been directly tested in military cohorts. Consequently, alterations of the gut-brain axis represent a plausible, but as yet unconfirmed, biological mechanism through which chronic circadian disruption may reduce physiological resilience in operational environments.

A submarine study found that the 6-h-on/6-h-off schedule led to fragmented, biphasic sleep during a 67-day undersea mission (Van Puyvelde et al., 2022). Melatonin did not adapt to the imposed work schedule, while cortisol showed greater adaptation to the work-sleep cycle. Overall, the findings demonstrate that human physiology cannot fully adapt to operational demands, highlighting the need for work schedules and countermeasures that better align operational requirements with biological circadian rhythms.

Hu and Liu (2023) investigated 278 Navy sailors to examine the impact of circadian rhythm disruption on health and operational capability. Their findings showed that circadian rhythm disturbances were significantly associated with increased sleep disorders, fatigue, and health problems, suggesting that circadian misalignment is a key determinant of well-being and may adversely affect naval operational performance. A study in U.S. Navy sailors demonstrated that individual differences in chronotype significantly influenced sleep quality, alertness, and psychological health under military operational conditions. These findings highlight that circadian misalignment may not affect all service members equally, as personnel whose endogenous circadian phase is mismatched with imposed duty schedules may be at increased risk of fatigue and impaired mental well-being (Harrison et al., 2021). Evidence from civilian populations indicates that individual chronotype substantially influences vulnerability to circadian misalignment. When work or social schedules are incongruent with an individual’s endogenous circadian phase, sleep quality deteriorates, daytime sleepiness increases, and symptoms of depression and anxiety become more prevalent. Circadian misalignment is also associated with low-grade systemic inflammation, including elevated levels of IL-6 and C-reactive protein; while these peripheral markers do not by themselves establish central neuroinflammation, they provide a plausible biological pathway that may link chronotype-dependent sleep disruption to impaired mental well-being and cognitive performance (Takahashi et al., 2018; Yang et al., 2023).

To make explicit which conclusions rest on direct evidence in military personnel and which are extrapolated from civilian research or experimental/preclinical models, Table 1 categorizes the studies discussed above by evidence level. As the table illustrates, direct military studies that combine objective circadian biomarkers (e.g., melatonin, cortisol, actigraphy-derived phase markers) with inflammatory or neuroimmune biomarkers and neurocognitive or affective outcomes within the same cohort remain scarce; this combination represents the principal evidence gap for the field, and much of the mechanistic reasoning linking circadian disruption to neuroinflammation in military personnel is necessarily inferred from civilian and preclinical work (Table 1.)

**Table 1:** Representative evidence from experimental/preclinical, military, and civilian studies.

Reference	Population	Circadian-relevant exposure assessed	Outcome measured	Evidence type
Ballesio et al. (2025)	Human experimental studies (meta-analysis)	Partial sleep deprivation; circadian phase not directly assessed	Peripheral inflammatory markers, including IL-6 and CRP	Civilian – human
Damato et al. (2022)	USAF T-6A instructor pilots	Operational flight duty; circadian phase not measured	Serum IL-6, TNF-α; fatigue	Military – human
Eum et al. (2023)	Experimental model	Circadian disruption	Gut barrier integrity; β-catenin/MMP-related pathway	Experimental – preclinical
Harrison et al. (2021)	U. S. Navy sailors	Chronotype and duty-schedule characteristics	Sleep quality, alertness, psychological health	Military – human
Hu and Liu (2023)	Navy sailors	Circadian rhythm disturbance assessed	Sleep disorders, fatigue, health problems	Military – human
LaGoy et al. (2022)	Active-duty military personnel	Time of day + simulated military operational stress	Perception–action coupling performance	Military – human
Lee et al. (2009)	Healthy young adults	Forced-desynchrony protocol (28-h day); circadian phase experimentally manipulated	Neurobehavioral and cognitive performance	Civilian – human
Li et al. (2014)	Military personnel undergoing intensive combat training	Intensive combat training; circadian misalignment not directly assessed	Serum S100B and immune-related measures	Military – human
Liechty et al. (2026)	Submariners	Combined circadian/sleep/light-cue disruption	Gut microbiota, mood, fatigue	Military – human
Pedersen and Hansen (2026)	Danish military pilots	Transmeridian flights; circadian disruption not directly measured	Prostate cancer incidence	Military – human
Skálová et al. (2026)	Military cadets	Total sleep deprivation; circadian rhythmicity assessed	Cognitive performance, mood	Military – human
Stark et al. (2024)	Female U. S. military aviation officers	Night operations, irregular schedules; circadian phase not measured	Reproductive health	Military – human
Teglas et al. (2023)	Experimental model	PCB exposure; endothelial circadian clock dysregulation	Endothelial circadian clock; endothelial toxicity	Experimental – preclinical
Teglas et al. (2024)	Experimental model	Circadian regulation under environmental toxicant exposure	Endothelial tight-junction integrity; mitochondrial bioenergetics	Experimental – preclinical
Van Puyvelde et al. (2022)	Submariners, 67-day mission	6-h-on/6-h-off schedule; melatonin and cortisol measured	Sleep fragmentation, hormonal rhythms	Military – human
Varanoske et al. (2022)	U. S. Marines undergoing SERE training	Severe operational/training stress; circadian misalignment not directly assessed	S100B, inflammatory/stress-related biomarkers, cognitive performance and mood	Military – human
Verissimo França de Oliveira et al. (2025)	Brazilian A-29 fighter pilots	Acute flight missions; circadian phase not assessed	Immune cell counts, metabolomics	Military – human

## Potential intervention strategies in the military

Circadian disruption represents a relevant neurobiological challenge in military populations, as operational demands frequently require performance during biologically adverse phases, prolonged wakefulness, and irregular sleep–wake schedules. Sunde et al. (2022) demonstrated that exposure to bright light during nighttime military operations improved subjective alertness, reduced sleepiness, and helped maintain performance. These findings support the use of appropriately timed light exposure as a potential operational countermeasure to support circadian adaptation and alertness. Similarly, a study of 42 active-duty U.S. Navy submariners demonstrated that individualized light therapy improved sleep quality, enhanced projected performance, and facilitated circadian adaptation during a 12-day operational deployment, highlighting its potential as a practical strategy for mitigating the effects of military-related circadian disruption (Chabal et al., 2024).

Strategic napping represents another potential countermeasure. Short, appropriately timed naps can reduce sleepiness and improve alertness and cognitive performance, including sustained attention and psychomotor vigilance, particularly under conditions of sleep restriction or extended wakefulness (Brooks and Lack, 2006; Signal et al., 2009). In military settings, brief nap opportunities may be particularly useful when consolidated sleep is operationally constrained. Indeed, a 30-min nap has been shown to mitigate performance deterioration following sustained military operations involving partial sleep deprivation (Keramidas et al., 2018). When operationally feasible, longer nap opportunities may provide additional restorative benefits, including improvements in cognitive performance, although sufficient time should be allowed after awakening to minimize the effects of sleep inertia (Boukhris et al., 2020; Oriyama, 2024). Thus, strategic napping may represent a practical approach to partially counteract sleep-loss-related decrements in alertness and performance during sustained operations.

The importance of maintaining adequate and consistent sleep is further supported by controlled experimental evidence demonstrating a dose-dependent decline in sustained attention, reaction time, and cognitive performance with progressively restricted sleep duration (Belenky et al., 2003). In this study, participants were assigned to different sleep-opportunity conditions, with scheduled time in bed restricted to 3, 5, 7, or 9 h per night for seven consecutive days. Performance in the 9-h group remained relatively stable, whereas participants in the restricted-sleep groups exhibited progressive performance impairments and incomplete recovery following subsequent recovery sleep. These findings support the potential value of protected sleep opportunities, stable sleep windows, and optimized duty–rest schedules in military environments.

Individual differences in circadian preference should also be considered when designing countermeasures. Individuals with an evening chronotype generally tolerate early start times less well, whereas those with a morning chronotype may experience greater difficulty adapting to night work. Chronotype can therefore influence adaptation to shift work (Kerkhof, 2018). Assessment using validated tools, such as the Morningness–Eveningness Questionnaire or Munich Chronotype Questionnaire, together with personalized scheduling where operationally feasible, may support more individualized approaches to managing circadian strain.

Finally, comprehensive education may represent a practical and low-cost component of circadian and sleep management. Increasing awareness of circadian rhythms, strategic light exposure, and sleep hygiene may facilitate the adoption of evidence-based behavioral strategies. A randomized trial among Royal Australian Navy sailors demonstrated that a brief educational program focusing on circadian rhythms, strategic light exposure, and sleep hygiene improved sleep quality and psychological health during at-sea deployments (Schmied et al., 2022). Together, these findings suggest that education may complement other operational countermeasures aimed at supporting sleep, circadian adaptation, and operational performance. Figure 1 summarizes potential operational strategies to support circadian alignment, sleep, alertness, and performance among military personnel.

**Figure 1:**
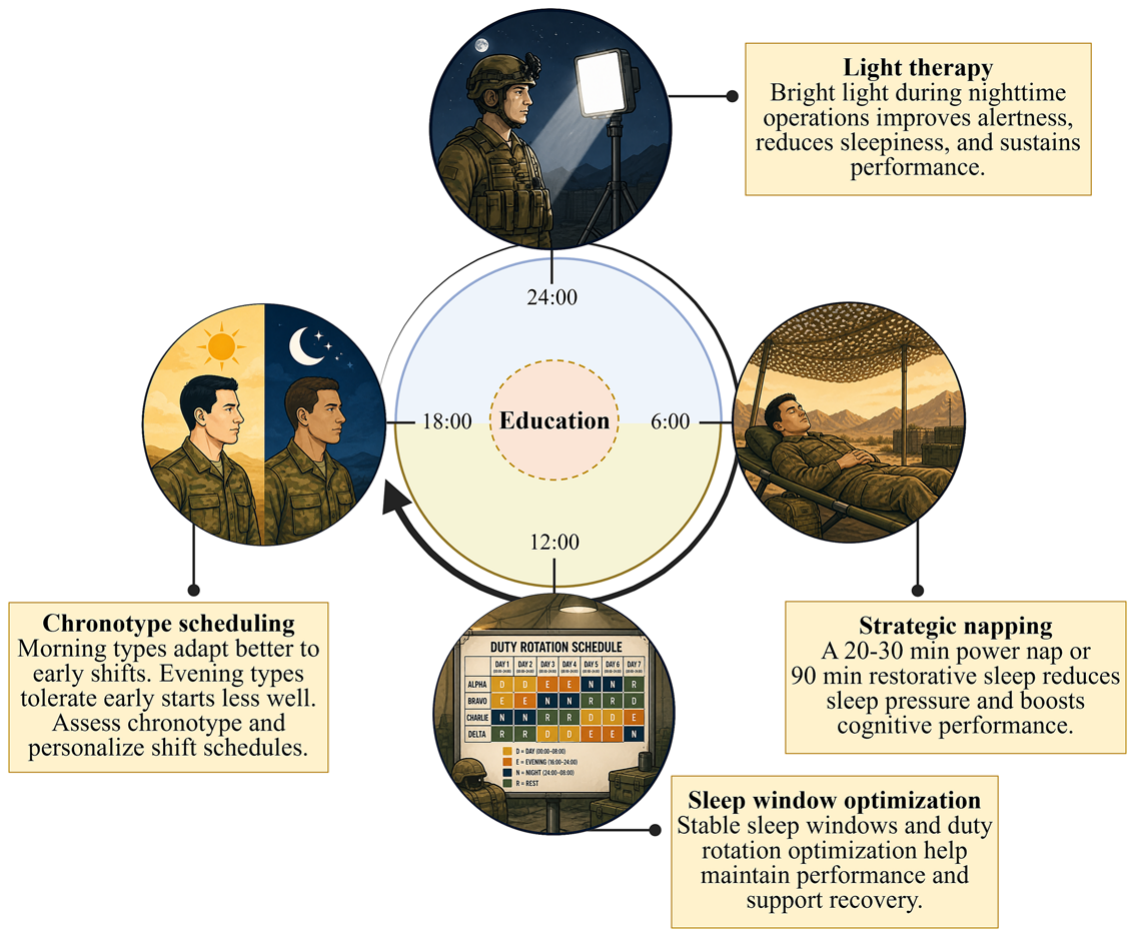
Potential operational strategies to support circadian alignment, sleep, alertness, and performance in military personnel.

## Limitations

Despite increasing interest in the relationship between circadian disruption, inflammation, and operational performance, several limitations of the current evidence base should be acknowledged. Much of the available literature is observational or based on short-term experimental studies, limiting conclusions regarding causality and long-term neurological consequences. Moreover, circadian disruption frequently co-occurs with sleep restriction, shift work, and operational stress, making it difficult to disentangle their independent effects. Inflammatory evidence is also predominantly based on peripheral biomarkers, which provide biological plausibility but do not directly demonstrate central neuroinflammation. Importantly, military populations are heterogeneous with respect to occupational demands, deployment conditions, schedules, and environmental exposures, which may contribute to variability across studies. Finally, direct evidence linking objectively measured circadian disruption to neuroinflammatory processes and neurocognitive or affective outcomes in military personnel remains limited. These limitations highlight the need for well-controlled, longitudinal studies integrating objective circadian measures with peripheral and, where feasible, central neuroimmune markers and functional outcomes.

## Conclusion

Circadian disruption may represent a potentially modifiable neurobiological factor associated with neuroinflammation in military personnel. Chronic circadian misalignment may contribute to neuroinflammation, cognitive impairment, mood disturbances, and reduced operational readiness. Targeted strategies, including timed light exposure, strategic napping, optimized sleep schedules, chronotype-based scheduling, and circadian education, may help mitigate these effects. Further research is needed to establish personalized, evidence-based interventions to protect brain health and operational performance.
